# The Behavior of Muscle Oxygen Saturation, Oxy and Deoxy Hemoglobin during a Fatigue Test in Fibromyalgia

**DOI:** 10.3390/biomedicines11010132

**Published:** 2023-01-04

**Authors:** Santos Villafaina, Pablo Tomas-Carus, Vanda Silva, Ana Rodrigues Costa, Orlando Fernandes, Jose A. Parraca

**Affiliations:** 1 Facultad de Ciencias del Deporte, Universidad de Extremadura, 10003 Cáceres, Spain; 2 Departamento de Desporto e Saúde, Escola de Saúde e Desenvolvimento Humano, Universidade de Évora, 7004-516 Évora, Portugal; 3 Comprehensive Health Research Centre (CHRC), University of Évora, 7004-516 Évora, Portugal; 4 Family Health Unit—Lusitania, Rua do Ferragial do Poço Novo, S/N, 7000-727 Évora, Portugal; 5 Departamento de Ciências Médicas e da Saúde, Escola de Saúde e Desenvolvimento Humano, Universidade de Évora, 7004-516 Évora, Portugal

**Keywords:** strength, mitochondrial, autonomic modulation, physical exercise, fatigue

## Abstract

Previous studies have reported that people with fibromyalgia (FM) could suffer from mitochondrial dysfunction. However, the consumption of muscle oxygen during physical exercise has been poorly studied. Therefore, this study aimed to explore the response of muscle oxygen during a fatigue protocol in people with FM and healthy controls (HC). In addition, the peak torque and the total work were assessed. A total of 31 participants (eighteen were people with fibromyalgia and thirteen were healthy controls) were enrolled in this cross-sectional study. All the participants underwent a fatigue protocol consisting of 20 repetitions at 180°·s^−1^ of quadriceps flexions and extensions using a Biodex System 3. The muscle oxygen saturation (SmO_2_), total hemoglobin (THb), deoxygenated hemoglobin (HHb) and oxygenated hemoglobin (O_2_Hb) values were measured using a portable near-infrared spectroscopy (NIRS) device. Significant differences between people with FM and healthy controls were found at baseline: SmO_2_ (FM: 56.03 ± 21.36; HC: 77.41 ± 10.82; *p* = 0.036), O_2_Hb (FM: 6.69 ± 2.59; HC: 9.37 ± 1.31; *p* = 0.030) and HHb (FM: 5.20 ± 2.51; HC: 2.73 ± 1.32; *p* = 0.039); during the fatigue protocol: SmO_2_ (FM: 48.54 ± 19.96; HC: 58.87 ± 19.72; *p* = 0.038), O2Hb (FM: 5.70 ± 2.34; HC: 7.06 ± 2.09; *p* = 0.027) and HHb (FM: 5.69 ± 2.65; HC: 4.81 ± 2.39; *p* = 0.048); and in the recovery at three min and six min for SmO_2_, O_2_Hb and HHb (*p* < 0.005). Furthermore, healthy control values of SmO2, O2Hb and HHb have been significantly altered by the fatigue protocol (*p* < 0.005). In contrast, people with FM did not show any significant alteration in these values. Moreover, significant differences were found in the peak torque at extension (FM: 62.48 ± 24.45; HC: 88.31 ± 23.51; *p* = 0.033) and flexion (FM: 24.16 ± 11.58; HC: 42.05 ± 9.85; *p* = 0.010), and the total work performed at leg extension (FM: 1039.78 ± 434.51; HC: 1535.61 ± 474.22; *p* = 0.007) and flexion (FM: 423.79 ± 239.89; HC: 797.16 ± 194.37; *p* = 0.005).

## 1. Introduction

Chronic, widespread and persistent pain is the most recognized symptom of fibromyalgia (FM) [1]. However, there are others, such as stiffness, depression, sleep disorders, mobility impairments and anxiety [1,2], that significantly impact the health-related quality of life (HRQOL) of people with FM [3]. FM affects 2.7% of the population, with a female/male ratio of 1.5:1, with women representing 58.7% of FM cases [4].

A previous study showed that HRQOL was significantly affected by physical, social and psychological factors [3]. In addition, recent studies showed significant differences in autonomic modulation, brain morphology, strength and neuromuscular impairments [5,6,7,8] between people with FM and healthy controls. People with fibromyalgia showed dysautonomia (autonomic nervous system hyperactivity at rest and hyperreactivity during stressful situations) [9,10,11,12,13]. Due to the connection between the autonomic nervous system and the cardiovascular system [14], these findings may be connected with the abnormal cerebral blood flow dynamics observed in people with FM [14,15] or the hemodynamics abnormalities reported by people with chronic fatigue and FM [16,17]. 

Physical exercise has demonstrated strong evidence against FM symptoms [18]. Previous studies have reported benefits in terms of HRQOL, physical function, pain and the brain’s electrical patterns after physical exercise interventions [19,20,21,22,23,24]. Despite the benefits, people with FM exhibited lower adherence to physical exercise. This could be due to the fact that 40% of people show fear of movement and avoidance behaviors regarding physical activity [25]. Thus, treatment adherence is conditioned by high pain levels [26]. In this regard, a previous study reported that >50% of the variation in pain intensity was explained by the metabolic situation and blood flow of the analyzed muscle (in this case, the trapezius and the erector spinae muscles) [27]. In line with these findings, Shang, Gurley, Symons, Long, Srikuea, Crofford, Peterson and Yu [17], using optical spectroscopies, showed an alteration of muscle oxygen utilization in people with FM while performing 6 sets of 12 isometric contractions of knee extensor muscles at 20 to 70% of their maximal voluntary isometric contraction. However, to the best of our knowledge, this is a unique study that investigates muscle oxygen utilization in people with FM. Nevertheless, since the study of the cardiovascular system and the autonomic nervous system during exercise can provide useful information in the prognosis of diseases [28], previous studies have analyzed the autonomic modulation during physical exercise interventions [29,30].

Therefore, there is a need for studies that investigate the consumption of muscle oxygen during physical exercise. For this reason, muscle hemodynamics and metabolism have been manipulated using an isokinetic fatigue protocol (consisting of 20 repetitions at 180°·s^−1^). Thus, our study aimed to explore the differences between people with FM and healthy controls on the consumption of muscle oxygen while performing an isokinetic strength fatigue protocol. We hypothesized that FM would significantly affect the normal metabolic muscle response observed in healthy controls. In addition, we hypothesized that the levels of work and peak torque performed by people with FM would be significantly lower than those achieved by people with FM.

## 2. Materials and Methods

### 2.1. Participants

G*Power software 3.1.9.4 (Kiel University, Kiel, Germany) estimated that a total sample size of eight women with FM achieves a 95% power to detect significant differences with an alpha of 0.005, using the Wilcoxon signed-rank test. The values of oxygen extraction fraction provided by a previous study [17] (99.7 ± 2.6 vs. 107.4 ± 2.0; *p*-value = 0.03) were used to make this calculation. Therefore, the recruitment objective was to include the greatest number of participants until April 2021. Thus, a total of 31 women (eighteen were women with fibromyalgia and thirteen were healthy control women) were enrolled in the study. The inclusion criteria were: (a) be diagnosed according to the American College of Rheumatology’s criteria [31], (b) be a female, (c) be able to communicate with the research staff, (d) have read and signed the written informed consent. Participants were excluded if they: (a) had contraindications for physical exercise, (b) suffered from a neurological disorder, such as Alzheimer’s disease, other vascular dementias, Parkinson’s disease, strokes, brain and vertebral tumors, multiple sclerosis and other dystonias, (c) suffered from diabetes mellitus or (d) were pregnant.

The mean age and body mass index (BMI) of people with fibromyalgia were 51.3 (10.4) years and 31.5 (7.9) kg/m^2^, respectively. For healthy controls, the mean age was 40.3 (1.8) years and the mean BMI was 22.6 (3.7). All of the women with FM, excluding two women, were under pharmacological treatment (mainly antidepressants, analgesics and muscle relaxants). Healthy controls were not under any pharmacological treatment.

The convenience sample was recruited from the Lusitania family health unit in Évora (Portugal) and the University of Évora until April 2021. All procedures were conducted following the Helsinki Declaration (revised in Brazil, 2013) and approved by the university’s research ethics committee (GD/44902/2019).

### 2.2. Procedure

A warm-up was performed for three minutes using a cycle ergometer (Monark Exercise AB, Vansbro, Sweden) at 50–60 rpm with no resistance to avoid fatigue. After that, three repetitions of knee extension and flexion were conducted with a Biodex System 3 (Biodex Corporation, Shirley, NY, USA). These repetitions were conducted with their dominant leg at free velocity and without load.

The fatigue protocol consisted of 20 repetitions of knee extension and flexion of the dominant leg at 180°·s^−1^ [32,33]. The peak torque at extension and flexion, as well as the total work at extension and flexion, were extracted. Measurements took place between 9:30 and 12:30 am and participants were encouraged to avoid any type of physical exercise before the protocol.

### 2.3. Instruments

#### 2.3.1. NIRS Sensor

Muscle oxygen saturation (SmO_2_), total hemoglobin (THb), deoxygenated hemoglobin (HHb) and oxygenated hemoglobin (O_2_Hb) values were measured using a portable NIRS sensor (Moxy, Fortiori Design LLC, Hutchinson, MN, USA) connected with GoldenCheetah software (version 3.4, U.S.). This device is reliable at low and moderate intensity for measuring consumption of muscle oxygen (SmO2; ICC: r = 0.773–0.992) [34]. The device was placed in the vast lateral quadriceps between the greater trochanter and the lateral femoral epicondyle. To reduce noise, a soft spline filter was applied using MATLAB^®^ software (MathWorks, Inc., Natick, MA, USA). We used a second-order 6Hz cut-off Butterworth filter, applied two times to the time series.

#### 2.3.2. Sociodemographic Data and Physical Activity Level

Age, duration of the disease, years since the diagnosis, work information and education level were asked of participants before the protocol. Moreover, participants were weighed and their heights were measured using a stadiometer (SECA 225, SECA, Hamburg, Germany). The 36-Item Short Form Health Survey questionnaire (SF-36) was used to evaluate the health-related quality of life (HRQoL) [35]. This questionnaire comprises eight domains (physical functioning, role physical, bodily pain, general health, vitality, social functioning, role emotional and mental health). The reliability of this questionnaire showed a Cronbach’s alpha greater than 0.85 [36]. This questionnaire has been previously used in people with FM [37]. The physical activity level was asked using the International Physical Activity Questionnaire (IPAQ). It is used to assess physical activity and time spent sitting [38]. This questionnaire has not shown higher test–retest reliability compared to objective measurements [39]. However, this questionnaire was only used to characterize the physical activity pattern of both groups.

### 2.4. Statistical Analysis

Statistical software SPSS (Statistical Package for Social Sciences, version 25) was used to perform the statistical analyses. Since a Mann–Whitney U test revealed significant differences at baseline between people with fibromyalgia and healthy controls in age and BMI, these two variables have been used as covariates in the between-groups analyses. In this regard, ANCOVA was used to explore between-groups differences. In addition, a Friedman test was conducted to evaluate within-group differences in fibromyalgia and healthy control groups. The eta partial square effect size was calculated and classified as follows: >0.5 is a large effect, between 0.5 and 0.3 is a medium effect, and <0.3 is considered a small effect [40,41].

## 3. Results

The Table 1 below shows the characteristics of the sample. Differences between people with fibromyalgia and healthy controls were found in age (*p*-value = 0.014) and BMI (*p*-value = 0.001). Thus, these two variables have been included as covariates in the between-groups analysis. In addition, differences were also observed in the HRQoL (*p*-value = 0.001), type of work (*p*-value = 0.009), education level (*p*-value = 0.009) and total minutes at vigorous (*p*-value = 0.003) and moderate (*p*-value = 0.007) exercise in a week.

Table 2 shows the differences between people with fibromyalgia and healthy controls in the consumption of muscle oxygen before, during and after the fatigue protocol. Significant differences were found at baseline in SmO_2_ (*p*-value = 0.036), O_2_Hb (*p*-value = 0.030) and HHb (*p*-value = 0.039). During the warm-up, differences were not found between people with fibromyalgia and healthy controls.

During the fatigue protocol, differences were found in SmO_2_ (*p*-value = 0.038), O_2_Hb (*p*-value = 0.027) and HHb (*p*-value = 0.048). In addition, a significantly different response in the consumption of muscle oxygen was observed during recovery at three and six minutes after the fatigue protocol in SmO_2_, O_2_Hb, and HHb (*p*-value < 0.005).

**Figure 1:**
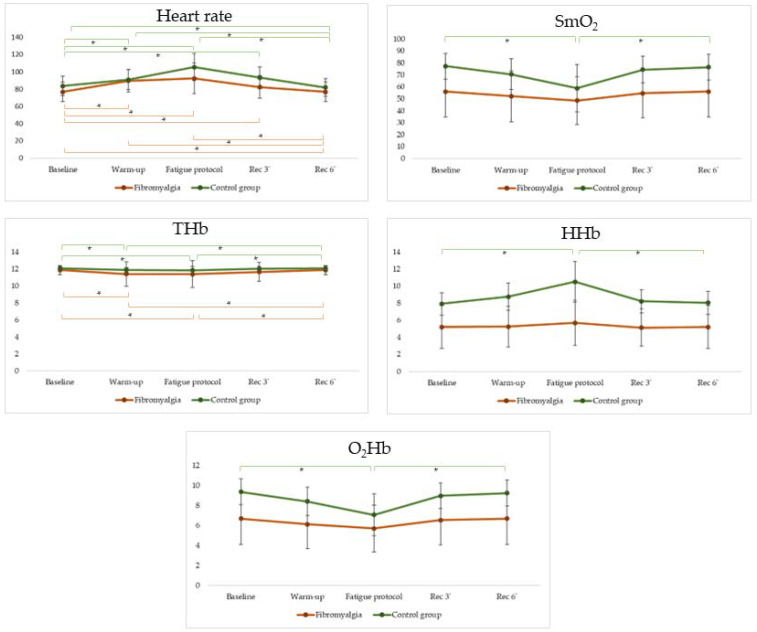
Evolution of consumption of muscle oxygen in people with fibromyalgia and healthy controls before, during and after a fatigue protocol. SmO_2_: muscle oxygen saturation; THb: total hemoglobin; HHb: deoxygenated hemoglobin; O_2_Hb: oxygenated hemoglobin; * *p* < 0.005.

Figure 1 shows the evolution of consumption of muscle oxygen in people with fibromyalgia and healthy controls before, during and after a fatigue protocol. Friedman tests for each group revealed significant differences for all variables studied (*p*-value < 0.005). Pairwise comparisons are detailed in Figure 1 for each variable.

Table 3 shows the differences between people with fibromyalgia and healthy controls in the isokinetic strength during a protocol consisting of 20 repetitions at 180°·s^−1^. People with fibromyalgia exhibited a significantly lower peak torque at extension (*p*-value = 0.033) and flexion (*p*-value = 0.010). In addition, the total work during the protocol was significantly lower at extension (*p*-value = 0.007) and at flexion (*p*-value = 0.005).

## 4. Discussion

This study aimed to explore the differences between people with FM and healthy controls on the consumption of muscle oxygen while performing an isokinetic strength fatigue protocol. We hypothesized that FM would significantly affect the normal metabolic muscle response observed in healthy controls. In addition, we hypothesized that the levels of work and peak torque performed by people with FM would be significantly lower than those achieved by people with FM. Results showed differences at baseline, during the fatigue protocol and in the recovery at three min and six min on SmO_2_, O_2_Hb and HHb between people with FM and healthy controls. In addition, differences were also observed in the evolution of consumption of muscle oxygen during the procedure between people with FM and healthy controls. For instance, whereas in healthy controls, values of SmO_2_, O_2_Hb and HHb have been significantly altered by the fatigue protocol, people with FM did not show any significant alteration. Moreover, significant differences were found in the peak torque and total work performed at leg flexion and extension.

Our results showed significant differences between people with FM and healthy controls in SmO_2_, O_2_Hb and HHb values. In this regard, healthy controls showed higher levels of SmO_2_ and O_2_Hb as well as lower values of HHb than people with FM. These differences can be observed even at rest. Similar results have been obtained in a previous study [17]. In addition, when the fatigue exercise protocol started, the consumption of muscle oxygen of healthy controls tended to decrease SmO_2_ levels and increase HHb levels due to the utilization of oxygen for energy production. However, muscle oxygen consumption patterns of people with FM significantly differed from healthy controls. In this regard, values of SmO_2_ and HHb tended to be stable during the entire duration of the fatigue protocol. Future studies should explore the impact of pharmacological and non-pharmacological therapies on the consumption of muscle oxygen in people with FM.

A previous study found a lower relative oxygen extraction fraction in people with FM during exercise [17]. The authors hypothesized that this finding could be related to an altered mitochondrial function in people with FM. The mitochondrial dysfunction could make energy production insufficient due to an abnormal synthesis of adenosine triphosphate (ATP). Therefore, muscle fatigue would increase [42] since exercise demands would not be achieved. Furthermore, mitochondrial dysfunction could cause oxygen debt to be higher in people with FM than in healthy controls [43,44,45,46]. The oxygen debt is manifested by an increase in muscle lactate concentrations due to anaerobic respiration. Interestingly, mitochondrial dysfunction as well as lactate accumulation have been related to pain [47] and fatigue in people with FM [42]. 

Previous studies have found that people with FM showed lower levels of strength than healthy controls [5,48,49]. In addition, Park et al. [50] showed that people with FM exhibited lower levels of phosphocreatine (PCr) and ATP during a repeated isometric quadriceps contraction protocol. Furthermore, Lund et al. [51] analyzed pH levels during a sub-maximal and maximal controlled dynamic activation of the forearm flexor muscle group. Authors [51] observed that people with FM experienced the same decrease in pH after performing half the amount of work of healthy controls. Thus, they hypothesized that impaired muscle metabolism and/or microcirculatory disorder might explain these results. In this regard, a previous study showed functional disturbances of microcirculation in people with FM and those morphological abnormalities may also influence their microcirculation [52]. Regarding microcirculatory disorders, people with FM showed autonomic dysfunction (dysautonomia). Dysautonomia could lead to blood flow abnormalities and, therefore, be associated with fatigue and pain.

Previous studies have shown that the autonomic nervous system (ANS) of people with FM remained hypoactive to exercise [53,54]. The normal response to exercise would be that, during exercise, the sympathetic activity increases vasoconstriction, and increases HR and myocardial contractility. In this regard, a previous study showed that people with FM exhibited higher sympathetic modulation than healthy controls during a fatigue protocol [53]. This behavior has also been found in previous studies [55,56]. Thus, the observed pattern in people with FM induced by autonomic nervous system dysfunction may lead to chronic blood flow abnormalities [16]. Therefore, dysautonomia could explain the differences between people with FM and healthy controls in the consumption of muscle oxygen. However, our results showed that HR was significantly impacted in people with FM. Similar results were obtained in inflammatory biomarkers. Bote et al. [57] showed that a single bout of exercise could improve the inflammatory status in FM patients, reaching values similar to those obtained by healthy controls. This is relevant since FM syndrome may include a systemic and local chronic inflammatory state accompanied by an altered stress response [3]. Therefore, future studies should explore the acute effects of exercise in pathophysiological-related factors to better understand the benefits of physical exercise as a therapy.

To the best of our knowledge, this is the first study analyzing the consumption of muscle oxygen using a MOXY during a fatigue protocol in people with FM. However, this study has some limitations that should be highlighted. In this regard, differences between groups were found in age and BMI. Thus, between-groups analyses have been corrected, through ANCOVA, using age and BMI as covariates. Nevertheless, results are supported by previous investigations in the field [5,58]. In relation to this, BMI and multi-medication are factors that can produce bias in the results. However, these factors are usually associated with FM and, therefore, it is difficult to isolate them from each other [59,60]. In addition, the relatively small sample size means that these results cannot be extrapolated to all women with FM. Furthermore, the menopause status of women included in this study was not considered. This could be important since estrogens modulate molecular pathways related to vascular and skeletal muscle function [61,62]. In this regard, the electromyographic signal of the participants was not measured. Such an assessment would provide interesting information about neuromuscular impairments in this population. Future studies are encouraged to include electromyographic assessments during fatigue protocol. Due to these limitations, results must be taken with caution. Thus, future studies should replicate our study controlling for age, BMI and menopause status. 

## 5. Conclusions

Healthy controls showed an alteration in the values of SmO_2_, O_2_Hb and HHb before, during and after the fatigue protocol, whereas people with FM did not show any significant alteration. In addition, people with FM showed lower peak torque and total work performed at leg flexion and extension than healthy controls. These findings could suggest that people with FM had a significant impairment in the consumption of muscle oxygen.

## Figures and Tables

**Table 1:** Characteristics of the sample.

Variable	Fibromyalgia Mean (SD)	Healthy Controls Mean (SD)	*p*-Value
Age (years)	51.3 (10.4)	40.3 (1.8)	0.014
BMI (kg/m^2^)	31.5 (7.9)	22.6 (3.7)	0.001
Duration of fibromyalgia symptoms (years)	12.5 (9)	-	-
Years since diagnosis (years)	7.7 (6.4)	-	-
Work			0.025
With physical load	10 (55.6)	3 (21.4)	
Without physical load	2 (11.1)	11 (78.6)	
Without work	6 (33.3)	0	
Education level, N (%)			0.009
Primary school	9 (50%)	0 (0%)	
Secondary school	7 (38.9%)	3 (21.4%)	
University	2 (11.1%)	11 (78.6%)	
Physical activity level			
Vigorous intensity (min)	43.3 (145.2)	158.5 (163.5)	0.003
Moderate intensity (min)	1443.8 (1054.7)	764.2 (1566)	0.007
Walking (min)	228.7 (268.1)	388.4 (627.6)	0.761
Sitting time (min)	152.1 (199.2)	293.1 (186.4)	0.573
HRQoL	0.58 (0.11)	0.82 (0.12)	0.001

BMI: Body mass index; HRQoL: Health-related quality of life.

**Table 2:** Differences in the consumption of muscle oxygen between people with fibromyalgia and healthy controls at baseline, during and after a fatigue protocol.

Variable	Fibromyalgia Mean (SD)	Healthy Controls Mean (SD)	*p*-Value	F	Effect Size
**Baseline**					
HR (bpm)	76.79 (11.45)	83.58 (11.48)	0.354	0.890	0.131
SmO_2_ (%)	56.03 (21.36)	77.41 (10.82)	0.036	4.879	0.153
THb (g/dL)	11.89 (0.54)	12.10 (0.26)	0.387	0.772	0.029
O_2_Hb (g/dL)	6.69 (2.59)	9.37 (1.31)	0.030	5.274	0.163
HHb (g/dL)	5.20 (2.51)	2.73 (1.32)	0.039	4.705	0.148
**Warm-up**					
HR (bpm)	89.68 (13.13)	90.96 (11.59)	0.739	0.423	0.045
SmO_2_ (%)	52.31 (21.31)	70.65 (13.02)	0.079	3.325	0.110
THb (g/dL)	11.41 (1.43)	11.91 (0.27)	0.677	0.178	0.007
O_2_Hb (g/dL)	6.13 (2.46)	8.40 (1.43)	0.062	3.804	0.123
HHb (g/dL)	5.25 (2.37)	3.51 (1.59)	0.093	3.042	0.101
**Fatigue protocol**					
HR (bpm)	92.46 (17.91)	105.49 (15.69)	0.499	0.471	0.018
SmO_2_ (%)	48.54 (19.96)	58.87 (19.72)	0.038	4.753	0.155
THb (g/dL)	11.40 (1.60)	11.86 (0.44)	0.901	0.016	0.001
O_2_Hb (g/dL)	5.70 (2.34)	7.06 (2.09)	0.027	5.517	0.175
HHb (g/dL)	5.69 (2.65)	4.81 (2.39)	0.048	4.287	0.142
**Recovery—three min**					
HR (bpm)	82.35 (13.15)	93.34 (12.61)	0.231	1.504	0.055
SmO_2_ (%)	54.65 (20.52)	74.47 (11.17)	0.049	4.281	0.141
THb (g/dL)	11.67 (1.11)	12.04 (0.25)	0.659	0.200	0.008
O_2_Hb (g/dL)	6.53 (2.46)	8.97 (1.28)	0.041	4.623	0.151
HHb (g/dL)	5.14 (2.19)	3.08 (1.36)	0.041	4.614	0.151
**Recovery—six min**					
HR (bpm)	76.79 (11.45)	81.92 (10.23)	0.436	0.627	0.024
SmO_2_ (%)	56.03 (21.36)	76.54 (10.82)	0.044	4.472	0.147
THb (g/dL)	11.89 (0.54)	12.08 (0.26)	0.425	0.656	0.025
O_2_Hb (g/dL)	6.69 (2.59)	9.25 (1.29)	0.037	4.818	0.156
HHb (g/dL)	5.20 (2.51)	2.84 (1.32)	0.048	4.307	0.142

HR: heart rate; SmO_2_: muscle oxygen saturation; THb: total hemoglobin; HHb: deoxygenated hemoglobin; O_2_Hb: oxygenated hemoglobin; g/dL: grams per deciliter.

**Table 3:** Differences in the isokinetic strength between people with fibromyalgia and healthy controls over 20 repetitions at 180°·s^−1^.

Variable	Fibromyalgia Mean (SD)	Healthy Controls Mean (SD)	*p*-Value	F	Effect Size
Peak torque at extension (N∙m)	62.48 (24.45)	88.31 (23.51)	0.033	5.086	0.929
Peak torque at flexion (N∙m)	24.16 (11.58)	42.05 (9.85)	0.010	7.733	1.412
Total work at extension (J)	1039.78 (434.51)	1535.61 (474.22)	0.007	8.564	1.564
Total work at flexion (J)	423.79 (239.89)	797.16 (194.37)	0.005	9.574	1.748

N∙m: Newton meter; J: Joules.

## Data Availability

Not applicable.

## References

[1] Wolfe F., Clauw D.J., Fitzcharles M.A., Goldenberg D.L., Katz R.S., Mease P., Russell A.S., Russell I.J., Winfield J.B., Yunus M.B. (2010). The American College of Rheumatology Preliminary Diagnostic Criteria for Fibromyalgia and Measurement of Symptom Severity. Arthritis Care Res..

[2] Núñez-Fuentes D., Obrero-Gaitán E., Zagalaz-Anula N., Ibáñez-Vera A.J., Achalandabaso-Ochoa A., López-Ruiz M.D.C., Rodríguez-Almagro D., Lomas-Vega R. (2021). Alteration of Postural Balance in Patients with Fibromyalgia Syndrome-A Systematic Review and Meta-Analysis. Diagnostics.

[3] Lee J.W., Lee K.E., Park D.J., Kim S.H., Nah S.S., Lee J.H., Kim S.K., Lee Y.A., Hong S.J., Kim H.S. (2017). Determinants of quality of life in patients with fibromyalgia: A structural equation modeling approach. PLoS ONE.

[4] Wolfe F., Walitt B., Perrot S., Rasker J.J., Häuser W. (2018). Fibromyalgia diagnosis and biased assessment: Sex, prevalence and bias. PLoS ONE.

[5] Bachasson D., Guinot M., Wuyam B., Favre-Juvin A., Millet G.Y., Levy P., Verges S. (2013). Neuromuscular fatigue and exercise capacity in fibromyalgia syndrome. Arthritis Care Res..

[6] Villafaina S., Collado-Mateo D., Fuentes-García J.P., Cano-Plasencia R., Gusi N. (2019). Impact of fibromyalgia on alpha-2 EEG power spectrum in the resting condition: A descriptive correlational study. BioMed Res. Int..

[7] Leon-Llamas J.L., Villafaina S., Murillo-Garcia A., Gusi N. (2021). Impact of Fibromyalgia in the Hippocampal Subfields Volumes of Women—An MRI Study. Int. J. Environ. Res. Public Health.

[8] Jacomini L.C.L., Silva N.A.D. (2007). Dysautonomia: An emerging concept in fibromyalgia syndrome. Rev. Bras. De Reumatol..

[9] Raj S.R., Brouillard D., Simpson C.S., Hopman W.M., Abdollah H. (2000). Dysautonomia among patients with fibromyalgia: A noninvasive assessment. J. Rheumatol..

[10] Solano C., Martinez A., Becerril L., Vargas A., Figueroa J., Navarro C., Ramos-Remus C., Martinez-Lavin M. (2009). Autonomic dysfunction in fibromyalgia assessed by the Composite Autonomic Symptoms Scale (COMPASS). JCR J. Clin. Rheumatol..

[11] Ulas U.H., Unlu E., Hamamcioglu K., Odabasi Z., Cakci A., Vural O. (2006). Dysautonomia in fibromyalgia syndrome: Sympathetic skin responses and RR interval analysis. Rheumatol. Int..

[12] Martinez-Lavin M. (2004). Fibromyalgia as a sympathetically maintained pain syndrome. Curr. Pain Headache Rep..

[13] Furlan R., Colombo S., Perego F., Atzeni F., Diana A., Barbic F., Porta A., Pace F., Malliani A., Sarzi-Puttini P. (2005). Abnormalities of cardiovascular neural control and reduced orthostatic tolerance in patients with primary fibromyalgia. J. Rheumatol..

[14] Montoro C.I., Duschek S., Schuepbach D., Gandarillas M.A., Reyes Del Paso G.A. (2018). Cerebral blood flow variability in fibromyalgia syndrome: Relationships with emotional, clinical and functional variables. PLoS ONE.

[15] Duschek S., Mannhart T., Winkelmann A., Merzoug K., Werner N.S., Schuepbach D., Montoya P. (2012). Cerebral blood flow dynamics during pain processing in patients with fibromyalgia syndrome. Psychosom. Med..

[16] Cook D.B., Stegner A.J., Nagelkirk P.R., Meyer J.D., Togo F., Natelson B.H. (2012). Responses to exercise differ for chronic fatigue syndrome patients with fibromyalgia. Med. Sci. Sport. Exerc..

[17] Shang Y., Gurley K., Symons B., Long D., Srikuea R., Crofford L.J., Peterson C.A., Yu G. (2012). Noninvasive optical characterization of muscle blood flow, oxygenation, and metabolism in women with fibromyalgia. Arthritis Res. Ther..

[18] Macfarlane G.J., Kronisch C., Dean L.E., Atzeni F., Hauser W., Fluss E., Choy E., Kosek E., Amris K., Branco J. (2017). EULAR revised recommendations for the management of fibromyalgia. Ann. Rheum. Dis..

[19] Busch A.J., Webber S.C., Brachaniec M., Bidonde J., Bello-Haas V.D., Danyliw A.D., Overend T.J., Richards R.S., Sawant A., Schachter C.L. (2011). Exercise therapy for fibromyalgia. Curr. Pain Headache Rep..

[20] Bidonde J., Busch A.J., Schachter C.L., Overend T.J., Kim S.Y., Góes S.M., Boden C., Foulds H.J.A. (2017). Aerobic exercise training for adults with fibromyalgia. Cochrane Database Syst. Rev..

[21] Júnior J.C.A., de Almeida Silva H.J., da Silva J.F.C., da Silva Cruz R., de Almeida Lins C.A., de Souza M.C. (2018). Zumba dancing can improve the pain and functional capacity in women with fibromyalgia. J. Bodyw. Mov. Ther..

[22] Gavi M.B.R.O., Vassalo D.V., Amaral F.T., Macedo D.C.F., Gava P.L., Dantas E.M., Valim V. (2014). Strengthening exercises improve symptoms and quality of life but do not change autonomic modulation in fibromyalgia: A randomized clinical trial. PLoS ONE.

[23] Collado-Mateo D., Dominguez-Muñoz F.J., Adsuar J.C., Merellano-Navarro E., Gusi N. (2017). Exergames for women with fibromyalgia: A randomised controlled trial to evaluate the effects on mobility skills, balance and fear of falling. PeerJ.

[24] Villafaina S., Collado-Mateo D., Fuentes J.P., Rohlfs-Domínguez P., Gusi N. (2019). Effects of exergames on brain dynamics in women with fibromyalgia: A Randomized controlled trial. J. Clin. Med..

[25] Nijs J., Roussel N., Van Oosterwijck J., De Kooning M., Ickmans K., Struyf F., Meeus M., Lundberg M. (2013). Fear of movement and avoidance behaviour toward physical activity in chronic-fatigue syndrome and fibromyalgia: State of the art and implications for clinical practice. Clin. Rheumatol..

[26] Lorente G.D., Stefani L.F.B.D., Martins M.R.I. (2014). Kinesiophobia, adherence to treatment, pain and quality of life in fibromyalgia syndrome patients. Rev. Dor.

[27] Gerdle B., Ghafouri B., Lund E., Bengtsson A., Lundberg P., Ettinger-Veenstra H.V., Leinhard O.D., Forsgren M.F. (2020). Evidence of Mitochondrial Dysfunction in Fibromyalgia: Deviating Muscle Energy Metabolism Detected Using Microdialysis and Magnetic Resonance. J. Clin. Med..

[28] Freeman J.V., Dewey F.E., Hadley D.M., Myers J., Froelicher V.F. (2006). Autonomic nervous system interaction with the cardiovascular system during exercise. Prog. Cardiovasc. Dis..

[29] Schamne J.C., Ressetti J.C., Lima-Silva A.E., Okuno N.M. (2021). Impaired Cardiac Autonomic Control in Women with Fibromyalgia is Independent of Their Physical Fitness. J. Clin. Rheumatol. Pract. Rep. Rheum. Musculoskelet. Dis..

[30] Kingsley J.D., Panton L.B., McMillan V., Figueroa A. (2009). Cardiovascular autonomic modulation after acute resistance exercise in women with fibromyalgia. Arch. Phys. Med. Rehabil..

[31] Wolfe F., Clauw D.J., Fitzcharles M.-A., Goldenberg D.L., Häuser W., Katz R.L., Mease P.J., Russell A.S., Russell I.J., Walitt B. (2016). 2016 Revisions to the 2010/2011 Fibromyalgia Diagnostic Criteria. Semin. Arthritis Rheum..

[32] Tomas-Carus P., Ortega-Alonso A., Pietilainen K.H., Santos V., Goncalves H., Ramos J., Raimundo A. (2016). A randomized controlled trial on the effects of combined aerobic-resistance exercise on muscle strength and fatigue, glycemic control and health-related quality of life of type 2 diabetes patients. J. Sport. Med. Phys. Fit..

[33] Clemente-Suárez V., Parraca J., Silva V., Batalha N., Costa A., Tomas-Carus P. (2021). Differences in Peripheral Vascular Response of a Fibromyalgia Patient in a Physical Fatigue Situation.

[34] Crum E.M., O’connor W.J., Van Loo L., Valckx M., Stannard S.R. (2017). Validity and reliability of the Moxy oxygen monitor during incremental cycling exercise. Eur. J. Sport Sci..

[35] Ware J.E. (1993). SF-36 Health Survey: Manual and Interpretation Guide.

[36] Brazier J.E., Harper R., Jones N.M., O’Cathain A., Thomas K.J., Usherwood T., Westlake L. (1992). Validating the SF-36 health survey questionnaire: New outcome measure for primary care. Bmj.

[37] Lins L., Carvalho F.M. (2016). SF-36 total score as a single measure of health-related quality of life: Scoping review. SAGE Open Med..

[38] Craig C.L., Marshall A.L., Sjöström M., Bauman A.E., Booth M.L., Ainsworth B.E., Pratt M., Ekelund U., Yngve A., Sallis J.F. (2003). International physical activity questionnaire: 12-country reliability and validity. Med. Sci. Sport. Exerc..

[39] Kaleth A.S., Ang D.C., Chakr R., Tong Y. (2010). Validity and reliability of community health activities model program for seniors and short-form international physical activity questionnaire as physical activity assessment tools in patients with fibromyalgia. Disabil. Rehabil..

[40] Fritz C.O., Morris P.E., Richler J.J. (2012). Effect Size Estimates: Current Use, Calculations, and Interpretation. J. Exp. Psychol. Gen..

[41] Coolican H. (2017). Research Methods and Statistics in Psychology.

[42] Cordero M.D., De Miguel M., Moreno Fernández A.M., Carmona López I.M., Garrido Maraver J., Cotán D., Gómez Izquierdo L., Bonal P., Campa F., Bullon P. (2010). Mitochondrial dysfunction and mitophagy activation in blood mononuclear cells of fibromyalgia patients: Implications in the pathogenesis of the disease. Arthritis Res. Ther..

[43] Yildiz Ş., Kiralp M.Z., Akin A., Keskin I., Ay H., Dursun H., Cimsit M. (2004). A new treatment modality for fibromyalgia syndrome: Hyperbaric oxygen therapy. J. Int. Med. Res..

[44] McIver K.L., Evans C., Kraus R.M., Ispas L., Sciotti V.M., Hickner R.C. (2006). NO-mediated alterations in skeletal muscle nutritive blood flow and lactate metabolism in fibromyalgia. Pain.

[45] Valkeinen H., Häkkinen A., Hannonen P., Häkkinen K., Alén M. (2006). Acute heavy-resistance exercise-induced pain and neuromuscular fatigue in elderly women with fibromyalgia and in healthy controls: Effects of strength training. Arthritis Rheum..

[46] Valkeinen H., Alén M., Häkkinen A., Hannonen P., Kukkonen-Harjula K., Häkkinen K. (2008). Effects of concurrent strength and endurance training on physical fitness and symptoms in postmenopausal women with fibromyalgia: A randomized controlled trial. Arch. Phys. Med. Rehabil..

[47] Katz D.L., Greene L., Ali A., Faridi Z. (2007). The pain of fibromyalgia syndrome is due to muscle hypoperfusion induced by regional vasomotor dysregulation. Med. Hypotheses.

[48] Maquet D., Croisier J.-L., Renard C., Crielaard J.-M. (2002). Muscle performance in patients with fibromyalgia. Jt. Bone Spine.

[49] Okumus M., Gokoglu F., Kocaoglu S., Ceceli E., Yorgancioglu Z.R. (2006). Muscle performance in patients with fibromyalgia. Singap. Med. J..

[50] Park J.H., Phothimat P., Oates C.T., Hernanz-Schulman M., Olsen N.J. (1998). Use of P-31 magnetic resonance spectroscopy to detect metabolic abnormalities in muscles of patients with fibromyalgia. Arthritis Rheum. Off. J. Am. Coll. Rheumatol..

[51] Lund E., Kendall S.A., Janerot-Sjöberg B., Bengtsson A. (2003). Muscle metabolism in fibromyalgia studied by P-31 magnetic resonance spectroscopy during aerobic and anaerobic exercise. Scand. J. Rheumatol..

[52] Morf S., Amann-Vesti B., Forster A., Franzeck U.K., Koppensteiner R., Uebelhart D., Sprott H. (2004). Microcirculation abnormalities in patients with fibromyalgia–measured by capillary microscopy and laser fluxmetry. Arthritis Res. Ther..

[53] Costa A.R., Freire A., Parraca J.A., Silva V., Tomas-Carus P., Villafaina S. (2022). Heart Rate Variability and Salivary Biomarkers Differences between Fibromyalgia and Healthy Participants after an Exercise Fatigue Protocol: An Experimental Study. Diagnostics.

[54] Adler G.K., Geenen R. (2005). Hypothalamic–pituitary–adrenal and autonomic nervous system functioning in fibromyalgia. Rheum. Dis. Clin..

[55] Staud R. (2008). Heart rate variability as a biomarker of fibromyalgia syndrome. Future Rheumatol..

[56] Martínez-Lavín M., Hermosillo A.G. (2000). Autonomic nervous system dysfunction may explain the multisystem features of fibromyalgia. Semin. Arthritis Rheum..

[57] Bote M.E., Garcia J.J., Hinchado M.D., Ortega E. (2013). Fibromyalgia: Anti-inflammatory and stress responses after acute moderate exercise. PLoS ONE.

[58] Lange E., Mannerkorpi K., Cider A., Archer T., Wentz K. (2017). Physiological adaptation in women presenting fibromyalgia: Comparison with healthy controls. Clin. Exp. Psychol..

[59] Fernandez-Feijoo F., Samartin-Veiga N., Carrillo-de-la-Peña M.T. (2022). Quality of life in patients with fibromyalgia: Contributions of disease symptoms, lifestyle and multi-medication. Front. Psychol..

[60] Ursini F., Naty S., Grembiale R.D. (2011). Fibromyalgia and obesity: The hidden link. Rheumatol. Int..

[61] Menazza S., Murphy E. (2016). The expanding complexity of estrogen receptor signaling in the cardiovascular system. Circ. Res..

[62] Enns D.L., Tiidus P.M. (2010). The influence of estrogen on skeletal muscle. Sport. Med..

